# A qualitative study exploring youth’s experiences of hospital- and integrated community-based mental health services: the YouthCan IMPACT initiative

**DOI:** 10.1186/s12888-025-07523-7

**Published:** 2025-11-12

**Authors:** Meaghen Quinlan-Davidson, J. L. Henderson, Peter Szatmari, Amy Cheung, Mahalia Dixon, Jacqueline Relihan, Dana Singer, Lisa D. Hawke, Kristin Cleverley

**Affiliations:** 1 Margaret and Wallace McCain Centre for Child, Youth and Family Mental Health, Centre for Addiction and Mental Health, Dalla Lana School of Public Health, University of Toronto, Toronto, Canada; 2 Margaret and Wallace McCain Centre for Child, Youth and Family Mental Health, Centre for Addiction and Mental Health, Department of Psychiatry, University of Toronto, Toronto, Canada; 3 Cundill Centre for Child and Youth Depression, Centre for Addiction and Mental Health, Department of Psychiatry, Hospital for Sick Children, Department of Psychiatry, University of Toronto, Toronto, Canada; 4 Department of Psychiatry, University of Toronto, Toronto, Canada; 5 Margaret and Wallace McCain Centre for Child, Youth and Family Mental Health, Centre for Addiction and Mental Health, Toronto, Canada; 6 Education Department, Collaborative Learning College, Centre for Addiction and Mental Health, Toronto, Canada; 7 Centre for Addiction and Mental Health, Department of Psychiatry, University of Toronto, Toronto, Canada; 8 Department of Psychiatry, Lawrence S. Bloomberg Faculty of Nursing and Faculty of Medicine, Centre for Addiction and Mental Health, University of Toronto, Toronto, Canada

**Keywords:** Youth, Mental health and substance use, Integrated collaborative care team model, Hospital-based care

## Abstract

**Background:**

Approximately 20% of Canadian youth (12–25 years) experience mental health and substance use (MHSU) concerns, with the majority not receiving timely evidence-informed care. The YouthCan IMPACT study was a pragmatic randomized controlled trial (pRCT) of an integrated youth services model. The pRCT compared integrated collaborative care teams (the intervention) to youth outpatient hospital psychiatric services (the treatment as usual control). We embedded a qualitative study within the pRCT to explore youth’s perceptions and experiences of the intervention and the control services.

**Methods:**

Youth participants (14–17 years) were recruited to the study within an ongoing pRCT from hospitals. Youth were purposively sampled from the broader pRCT sample based on sex assigned at birth and pRCT arm (intervention or control group). To capture service experience across a range of follow-up intervals, youth were eligible to participate in the study if they had completed a six-month or a 12-month assessment within the pRCT. Semi-structured interviews were conducted with *n* = 44 youth between January 2018 and December 2019 (*n* = 22 intervention and *n* = 22 control participants). The interview focused on access; needs and preferences; decision-making; and satisfaction, among other domains. Two coders thematically analyzed the groups separately. Coding differences were resolved through discussion with the research team.

**Results:**

Participants in both study arms articulated their service experiences around the themes of dignity, respect, and autonomy in care. Participants felt valued and respected when they receive tailored, consistent services; comprehensive information; were able to develop a supportive, collaborative relationship with the service provider; and used their voice in their care plan. They reported improved satisfaction, improvement in mental health status, and usefulness of services. Some youth within the hospital setting experienced services that were inappropriate, inefficient, and poorly coordinated. These experiences adversely affected youth’s dignity, respect, and autonomy.

**Conclusions:**

This study is the first embedded qualitative study globally within a pRCT that explores experiences of an integrated collaborative care team model and standard hospital care. The study adds to the limited evidence base on dignity, respect, and autonomy within youth MHSU services. Determining how youth experience services in community-based and hospital settings is critical as it helps reduce treatment gaps.

**Trial registration:**

Clinicaltrials.gov NCT02836080. Registration date 2016–07-14

**Supplementary information:**

The online version contains supplementary material available at 10.1186/s12888-025-07523-7.

## Background

Mental health and substance use (MHSU) symptoms largely emerge during childhood, adolescence and young adulthood [1–4]. Global estimates suggest that by 25 years of age, 62.5% of mental health disorders have appeared [5]. These disorders are the largest contributors to the disease burden in this age group and are characterized by relapse and chronicity with youth often not receiving the care they need [6]. Youth’s health needs are conceptualized as the interaction between mental health concerns, the social determinants of health, and individual risk and protective factors [4]. Indeed, although the persistence and severity of these disorders can be reduced through the implementation of early intervention and prevention efforts [7–10], youth often experience a delay in access to quality evidence-informed services, and only the minority receive specialized treatment services [11], contributing to a larger burden of disease [9, 12, 13]. In particular, treatment delay is associated with impaired functioning, delayed achievement of developmental milestones, poorer quality of life, and diminished economic productivity [1–4].

In Canada, youth (12–25 years) have described the mental health system as fragmented, insufficient, and ineffective [11]. Several barriers to youth mental health services have been identified, including long waitlists, poorly coordinated care, limited resources, and services not matching youth MHSU needs [14, 15]. At the same time, MHSU services are not always effective in treating or reducing youth MHSU, limited in part by not including the perspectives of youth and families [16] in the development of services. Access barriers and limitations in effectiveness influence the provision, uptake, quality and continued use of MHSU services by youth [17].

Within the past two decades, various interventions and models of care have been developed to address youth MHSU concerns [18], with studies showing the benefits of providing access to integrated models of service delivery for youth, like headspace in Australia and Jigsaw in Ireland [19–24]. These service models provide MHSU, primary health care, social and vocational services in one location to address youth’s needs [20, 21]. Components of these models include youth and family engagement and service co-design within community settings; prevention and early intervention services; strengths-based approaches; tailored care approaches; expedited access to psychiatric consultations; and evidence-informed practices [19, 21, 25–27]. Yet, despite the uptake and promotion of these models globally [27], there is a dearth of evidence on how these services are implemented within real-world service settings; how the services respond (if at all) to the needs of youth and families; and the effectiveness of such services [18, 21]. Furthermore, there is a lack of understanding of how youth experience this model of care and standard hospitalized care [21] in terms of mental health outcomes [28].

The YouthCan IMPACT initiative in Toronto, Canada aimed to provide accessible, youth friendly, integrated care tailored to the needs of youth [28, 29]. The initiative was co-designed by youth and families [30], and included a variation of these integrated services for youth – the integrated collaborative care team (ICCT) model within a pragmatic randomized controlled trial (pRCT) [21]. The pRCT compared ICCT (the Intervention) to treatment as usual in outpatient hospital psychiatric services (the Control) [21, 28, 29], and included an embedded qualitative study. To our knowledge, this study is the first embedded qualitative study within a pRCT globally that explores youth’s service experiences of ICCT and standard hospital care.

There are several benefits associated with embedding qualitative methods within RCTs. Although the “gold standard” for intervention evaluation, RCTs do not obtain an in-depth understanding of service successes and failures from the participant’s perspective [31]. Qualitative research provides rich insight into how and why services are (or are not) effective, the acceptability of the intervention, and how it may influence the health and wellbeing of participants [32]. These perspectives can be used to inform service policy and practice. The current qualitative study explores the perceptions and experiences of youth in terms of access; service setting and connection; fit of services, integration, and holistic needs; and empowerment, engagement, and parental involvement in the intervention and the control services within the YouthCan IMPACT initiative. The study was exploratory in nature. Following O’Cathain’s (2018) [33] recommendations on qualitative research within RCT, we conducted qualitative research to increase the evidence’s relevancy by listening to youth and understanding the complexity of the health services. As such, there were no a priori hypotheses. By investigating youth’s service experiences, this study contributes to a better understanding about the extent to which the models of care meet youth’s needs, from their perspective. It will also help inform services to better respond to youth needs and promote positive outcomes [34].

## Methods

This qualitative study follows similar methodology of embedding qualitative research in RCTs as described by Kaptchuk et al. (2009) [35]. Participants were recruited to the qualitative study within an ongoing pRCT as described below.

### The parent study

Study participants (14–17 years of age) were recruited into a pRCT from five Toronto, Ontario hospitals between 2016 and 2020 (Trial ID: NCT02836080) [21]. The hospitals included the Centre for Addiction and Mental Health (CAMH), the Hospital for Sick Children (SickKids), Sunnybrook Health Sciences Centre (SHSC), Michael Garron Hospital (MGH), and North York General Hospital (NYGH). Youth within each site (total *n* = 247) were randomized to either the control or intervention group. If randomized to the control, they received services at the hospital to which they were referred, which was one of five control group outpatient hospital adolescent psychiatry services (CAMH, SHSC, SickKids, MGH, and NYGH). If randomized to the intervention group, participants were assigned to receive services at their choice of one of three intervention group sites (Central Toronto, Scarborough, and Toronto East). Youth outcomes were assessed at baseline, 6 months, and 12 months. The study was approved by the Research Ethics Boards at CAMH; Sunnybrook; Michael Garron Hospital; SickKids; and North York General; each intervention site also provided organizational ethics approval. A detailed description of the methods for the pRCT are described elsewhere [21, 28].

#### Intervention

Youth randomized to the Intervention group received access to mental health care providers (e.g., social worker, psychiatrist, and nurse practitioner). As part of the intervention, they also received access to primary care providers, care navigators to coordinate care, and peer support workers at the three community-based sites. At the start of intervention services, all participants were offered rapid access to needs assessment and Solution-Focused Brief Therapy (SFBT), to identify their needs and preferences, to inform their service plan, as well as to ensure their immediate concerns were understood and addressed [36, 37]. SFBT is a strengths-based, evidence-informed treatment approach that supports the identification of internalizing, and externalizing concerns, including substance use, guiding the youth towards solutions to address those concerns [37, 38]. Using clinical assessments and a stepped-care approach, youth and families were subsequently provided with options to care and higher or lower intensity services based on their needs [39]. Further information about the trial is described elsewhere [28].

Youth randomised to the Control group received standard outpatient adolescent psychiatry services at one of five hospitals. These services were generally led by a psychiatrist and involved assessing the participant symptoms and developing a treatment plan, which may have included psychotherapy, medication, and referral onto other services. While outpatient hospital-based psychiatric services encourage a collaborative process in treatment plan development, youth did not co-develop their treatment plan. Information on treatment planning within the Control group has been published elsewhere [28].

#### Study setting and participants

Participants for the qualitative study were purposively sampled sequentially on the basis of sex assigned at birth (male, female) and pRCT arm (Intervention, Control group), following participant composition in the parent study between the intervention and control arms. Following the parent study [28], sex assigned at birth was selected as the variable for purposeful sampling, as we assumed that the most common mental health disorders would be anxiety and depression and that there would be sex differences associated with these conditions. Youth were eligible to participate in the qualitative study if, within the pRCT parent study, they had either 1) completed a six-month outcome assessment within three months of study recruitment, or 2) completed a 12-month outcome assessment and received services within the past six months within the intervention or control sites. This criteria was followed to ensure the breadth of experiences within the pRCT were captured. Essentially, we wanted to capture youth’s service experiences long-term, across a range of follow-up intervals. Data from the outcome assessments were not intended to inform the qualitative study. Youth were contacted by their preferred method (e.g., email, text, telephone) by a research team member, provided information on the qualitative study, and invited to participate. Qualitative interviews were conducted with *n* = 44 youth: 22 youth from the intervention group and 22 the Control group. Adapting principles by Francis et al. (2010) [40], we stopped recruiting once saturation was reached, in that we iteratively analysed the data until no further data was generated from the interviews that would meaningfully contribute to the research objectives. Participants were not blind to the treatment allocation.

#### Consent and interview procedure

Participation was voluntary. Prior to conducting the semi-structured interviews, consent was obtained. A member of the research team read aloud the consent form at the beginning of the interview, asked participants if they understood the consent process and if they had any questions. Research team members emphasized that participant responses would have no impact on their current or future use of MHSU services.

Semi-structured interviews were conducted between January 2018 and December 2019. The interviews lasted about an hour and were conducted over the phone. The objectives of the study were explained to each participant. Interviews were facilitated by a research staff trained in semi-structured interviewing. Each session was audio-recorded; participant names were not recorded or transcribed, with single identifiers of sequential numbers for each participant used.

A semi-structured interview guide was developed by the research team, including youth with lived experience, to focus on understanding youth service experiences. The interview guide was the same for both the Intervention and Control arms. Questions covered study timeline and overall experience; access to services; service setting and connection; fit of services, integration, and holistic needs; and empowerment, engagement, and parental involvement. The semi-structured interview guide can be found in the Additional File 1. Participants received a $50 gift card as honorarium for participating in the interviews.

## Analysis

All interviews were transcribed verbatim. The analysis followed the process recommended by Fereday et al. (2006) [41], to rigorously and inductively investigate youth’s perspectives and experiences while also developing a codebook as a way to organize and interpret the findings.

Interviews were read through twice and a draft coding framework was developed for both arms. The team analysed the Intervention interviews first. For the Control interviews, we iteratively refined the Intervention codebook based on additional codes generated from the Control interviews. The Intervention and Control findings were analysed separately. Given that we explored service experiences and perspectives overall, results were integrated and reported together during the write-up phase. NVivo 12 qualitative data analysis software [42] was used to independently code six interviews by MQD and a second coder for both arms. To ensure consistent use of the coding framework, intercoder reliability was checked for both arms. The research team met to discuss differences when identified, further refining code definitions and usage, and adapting the framework through consensus. For the Intervention group, the coders achieved 88% agreement (kappa = 0.76) indicating substantial agreement; for the Control group, the coders achieved 92% agreement (kappa = 0.81) indicating almost perfect agreement [43]. The remaining interviews were coded in NVivo 12 [42] by arm by one researcher (MQD). The process involved the identification of common words and phrases, which were coded and later grouped into subthemes and themes. Following identification of key themes, the transcripts were coded according to the themes. As the data were reviewed, new themes and subthemes emerged and were adapted in an iterative process [44, 45]. Using a participatory action research approach [30], meetings were held with the youth co-researchers (MD, JR, DS) to review and revise the themes and subthemes to ensure that the findings reflect the lived experiences of youth, with minor edits made to the themes. The youth co-researchers did not code the data. Where appropriate, selected verbatim anonymised quotes from the participants were used to illustrate the themes identified in the study. Study participants’ medical records were also reviewed (with consent) to determine the number of visits youth had during the study and in relation to the qualitative interviews to contextualize their service experiences and perspectives.

## Results

### Participant characteristics

Demographic characteristics of youth participants were generally balanced across treatment conditions (Table 1). At the time of pRCT study enrolment, participants’ age ranged from 14 to 17 years. Further information about enrolment has been published elsewhere [46].

**Table 1:** Demographic characteristics of participants by intervention and control arm (*n* = 44)

	Intervention (*n* = 22)		Control (*n* = 22)		All (*n* = 44)	
	Mean	Range	Mean	Range	Mean	Range
Age	15.8	14–17	15.4	14–17	15.6	14–17
	**n**	**%**	**n**	**%**	**n**	**%**
**Gender Identity**						
Girl/Woman	15	77.3	18	81.8	33	75
Boy/Man	7	31.8	4	18.2	11	25
Transgender, non-binary	0	0	0	0	0	0
**Education Level**						
Grade 8 or less	2	9.1	5	22.7	7	15.9
Grade 9	5	22.7	4	18.2	9	20.4
Grade 10	11	50	8	36.4	19	43.2
Grade 11	3	13.6	4	18.2	7	15.9
High School Diploma/Trade/Certificate	1	4.5	1	4.5	2	4.5
**Employment Status**						
Unemployed	13	59.1	13	59.1	26	59.1
Part-Time	2	9.1	5	22.7	7	15.9
Volunteering	4	18.2	4	18.2	8	18.2
Co-Op Placement/Other	2	9.1	-	-	2	4.5
**Born in Canada**						
Yes	19	86.4	20	90.9	39	88.6
No	3	13.6	2	9.1	5	11.4
**Ancestry/ethnic group or cultural background**						
White	13	59.1	11	50	24	54.5
Asian	2	9.1	5	22.7	7	15.9
Black	3	13.6	0	0.0	3	6.8
Latin American	1	4.5	3	13.6	4	9.1
Another ethnicity (West Indian, Middle Eastern, Mixed)	3	13.6	3	13.6	6	13.6
	**Median**	**Range**	**Median**	**Range**	**Median**	**Range**
Number of service visits	3	0–40	5	0–50	5	0–50

Based on chart review data [46], the majority of Intervention group participants (16 youth) were interviewed after they had ceased to access services within this study, and six participants were interviewed while still using services during the study. Meanwhile, ten interviews took place after control group participants had ceased to access services within this study, ten interviews while youth were still using services, and one interview before any services had been received during the study (included as they had completed a six-month outcome assessment and were waiting to receive services).

Thematic analysis identified themes related to dignity and respect, as well as autonomy in care. See Additional File 2 for a list of exemplar quotes.

## Theme 1. Dignity and respect

Descriptions focussed on dignity and respect in a number of domains: service organization and navigation; privacy and confidentiality; compassionate support; and comfort.

### Subtheme 1. Service organization and navigation

Dignity and respect were described as maintained when youth received information about services, and experienced services and coordinated care that met their needs. Descriptions focused on service provider’s communicating to youth about the organization of services, youth’s preferences for how services are organized, and their experiences (or not) of coordinated care.

Participants in both study arms shared how comprehensive information on service access, process, and options to care were available to them. Youth were informed about options to care and resources (e.g., individual or group therapy), additional MHSU or educational services, and how to access support between sessions (e.g., service providers to contact). Being well-informed facilitated youth’s ability to ask questions, comfortably discuss their MHSU concerns with service providers, make decisions, feel prepared and reassured, and receive additional support through referral services and seamlessly navigate services.

Well I had meetings with the social worker, and we outlined all the services and programs they have there. And the different methods, everything that I really needed to know. And then I could pick from there which services I wanted to go forward with (Participant 21, Intervention Group, Boy/Man, 16 years of age).

Youth in both study arms described how providers communicated information, helped them navigate services, and instilled in them a sense of knowledge and competency to lead and be part of their care plan. It led to greater levels of trust and satisfaction with the services.

It’s never really been hidden from me at all how things work around here, which I really appreciated. That helps a lot with establishing that trust. But I just feel super comfortable being here and talking to people (Participant 14, Control, Female, 15).

When we were going over what services I would be put into, they gave me a whole list, like a whole list of programs and what they cover. So my provider felt that it would be better if I was put into a group therapy that goes over, like basically depression and anxiety. And so far, my experience in going to that service has been just every – Like it’s just gone over everything that I’ve been trying to work on over the past couple years. It made me very happy considering that it’s been a long time that I’ve been trying to find, like- trying to find a program that would fit these needs … and [this hospital] just had that program … (Participant 5, Control group, female, 14 years of age).

About half of the Control group participants experienced service disorganization and poor coordination of care. These youth acknowledged not knowing what services and resources were available to meet their needs, yet wanting information about service access, processes and options to care. They were uncertain about how they could engage and felt dismissed, sharing that the service was ‘a waste of their time’. These participants expressed uncertainty, frustration, distress, and lower levels of satisfaction.

Well at the end I just felt like I wasted my time there, because as, like I said, I live really far and they just took really long in general … Like, even though I received a service from them, like, I wasn’t satisfied with how it was really handled … because it was just – it was just way too long. And, it was – At the end like, I don’t know what I was trying to – trying to get from visits or receive at the end, but it just, like I just didn’t – all I know is that the doctor recommended me to come here and then I came and then it was just … really useless (Participant 3, Control, Girl/Woman, 16, table 3 for expanded quote)

From the chart review, these youth saw a service provider once or twice within the Control arm. These youth described how they did not receive information on next steps or follow-up on their care, or an explanation as to why they did not receive information. Some youth attributed this experience to their level of perceived need not being severe enough for the services.

But what I’ve been told is like … it’s not like severe enough for me to be placed high on waiting lists … ‘cause I’m like in that like middle zone where like everything is kind of shit, but it’s not like shit enough for me to like be given priority (Participant 40, Control, Girl/Woman, 17 years of age, table 3 for expanded quote).

At the same time, some Control group participants reported not feeling like equal partners in their care plan due to lack of information sharing and service disorganization. Youth described differential power dynamics and confusion. Youth also did not feel prioritized and were unsatisfied with the services received.

Based on the organization of services, youth in both arms described receiving services that met their needs (or not). There was a preference for receiving individualized, consistent care, however, youth in both study arms experienced barriers to this care. For some Intervention group participants, the main barrier was the facility’s walk-in model and the service’s preference for youth to access care in this manner. This barrier made youth’s ability to see the same provider at each visit challenging and in some cases prevented them from continuing care.

When I did need them it was hard … I didn’t feel comfortable enough to just be able to just go in there and then talk about this to someone I didn’t really know (Participant 26, Intervention, Girl/Woman, 16, table 3 for expanded quote).

Meanwhile, youth in the Control group reported that the lack of consistency in individualized care was due to the unavailability of this service option, particularly for those who felt that their MHSU concerns were not deemed severe enough.

Obviously they deal with much more severe cases, but at the same time … I would have liked it to be more – kind of – for me? (Participant 25, Control, Girl/Woman, 14, table 3 for expanded quote).

Similarly, some youth in the Intervention group were unable to access psychiatric care either due to the unavailability of the psychiatrist or referral and wait times to external psychiatric services. Although these youth expressed satisfaction at having some of their MHSU needs met, there was a need for a psychiatric evaluation that was unmet.

I went to [the Intervention site] for more of a traditional full psychiatric re-evaluation … Like, I wish it had gotten like more technical and more clinical in that regard (Participant 15, Intervention, Boy/Man, 16, table 3 for expanded quote).

According to participants, Intervention group providers coordinated care for youth. With the Control group, however, participants detailed how they were responsible for coordinating their own care. Some Control group participants detailed frustration at having to coordinate and follow-up themselves (or their caregiver) on referral services, as well as confusion as to why they were referred to external services, when they would have preferred to receive co-located services at the Control sites. This self-coordination of care led to treatment delay, as participants described waiting months for appointments at the referral service. These participants would have preferred greater information, direction, support resources, and coordination of care from Control sites.

It was frustrating because, I was like waiting … and it was like, “Oh, you gotta wait. Deal with your own problems for two more months.” (Participant 7, Control, Girl/Woman, 15, table 3 for expanded quote).

### Subtheme 2. Privacy and confidentiality

Participants shared how their privacy and confidentiality were maintained (or not) when using services in both study arms. When privacy and confidentiality were maintained, participants were satisfied and described feeling relief, comfortable, and safe.

Participant descriptions of privacy and confidentiality focused on the caregiver. Participants in both study conditions detailed how service providers reiterated privacy and confidentiality during sessions, asking the participant if they would like the caregiver to be present and involved in their session; requesting guidelines on what the youth would like addressed when the caregiver was present; and not divulging information to caregivers without the youth’s permission.

Participants in both study arms described how service providers also took steps to send password protected forms and materials to participants so that only the participant could access these resources at home. Wherein participants informed their service provider that their caregiver was not involved in their lives, the service took steps to coordinate care solely with the participant. According to participants, these steps included contacting the youth for appointments instead of the caregiver; making sure that other service referrals were aware of the youth’s confidentiality instructions; and ensuring that the youth could access medication independently. Some participants within the Control group discovered their caregivers had been contacted for appointment dates, despite explicitly asking the service to contact the youth directly; which led to missed appointments.

I think one of the biggest issues was that they tried to contact my mom specifically and that they didn’t go through me as well … (Participant 35, Control, Boy/Man, 17, table 3 for expanded quote).

Providers also sought permission from the youth to consult with other providers on their care plan. In both study arms, participants expressed satisfaction at sessions taking place behind closed doors, with white noise machines used to maintain privacy. According to youth, these actions facilitated their willingness to speak about their concerns.

Privacy for several participants in both study arms also meant not having to repeat their story to numerous providers. Repeating their story created distress, frustration and re-traumatization for youth in both study arms. It also adversely affected their continuity of care, as some participants described not returning to the service because they were unwilling to repeat their story.

I don’t know, like, I think not a lot of people like to go and retell their story every time … cause that’s really hard to do. (Participant 12, Intervention, Girl/Woman, 16, table 3 for expanded quote).

### Subtheme 3. Compassionate support

Participants in both study arms detailed how they felt cared for, not judged, valued, listened to, supported, safe, and comfortable when receiving services.

It made me feel like, you know like, everyone wants the best for me, like, you know, they want me to feel comfortable … (Participant 28, Intervention, Boy/Man, 15, table 3 for expanded quote).

Participants described how experiencing this support facilitated expressing their needs; and contributed to the continuation of care, trust, increased confidence, and satisfaction with services.

Just because like I’ve never been anywhere else and that was the place where I went to for a couple of months, weekly. Well, like, it really helped with my depression. It helped a lot. Because I was probably depressed for like a year, and now I feel like more like myself. (Participant 4, Intervention, Girl/Woman, 17)

In both study conditions, participants highlighted not feeling alienated, an outsider, or a burden. Experiencing this support empowered participants, reduced their distress, and motivated them to continue care.

I felt like they were actually talking to actually me as opposed to a person with problems or just like a patient. They made the effort to holistically take in everything that I was going through like my feelings and who I was with, like all that, to help me feel more comfortable and more willing to speak to them about my issues. (Participant 16, Intervention, Girl/Woman, 16 years).

Good. It makes me feel like, I’m going to someone who actually really cares about me, I guess. (Participant 27, Control, Girl/Woman, 16 years)

### Subtheme 4. Comfort

Participants articulated comfort as service provider commitment and a supportive setting.

#### Service provider commitment

Participants in both study arms described how health care providers were engaged or committed to their care. This commitment was detailed as building a bond with the provider; the availability of the provider outside of formal meetings; the provider’s knowledge and competency; and experiencing a tailored, individualized approach to care. When participants perceived that health care providers were committed to their treatment and care plan, youth expressed satisfaction and improvement in their mental health.

… it motivates me to actually form a good connection … she would ask, ask me questions and adapt to my needs it made me feel better knowing that she cares (Participant 37, Intervention, Girl/Woman, 17, table 3 for expanded quote).

In building a bond with the provider, participants from the Intervention sites described feeling understood, supported, and having their needs met. Participants described how they felt as though they were ‘talking to a friend’.

I was there to find some way to help with the things I was doing. To help find work around methods to manage and deal with my anxiety and self-loathing and all that. And I got exactly what I needed. I got help. I got–I got–I got strategies, methods and ways of dealing with my own personal problems. (Participant 36, Intervention, Boy/Man, 17, table 3 for expanded quote).

This relationship was facilitated by connecting with a service provider of similar gender identity and closeness in age. Some participants in both study conditions expressed a desire to be able to choose their provider based on gender identity, illustrating the importance of providing youth provider options.

… it would have been nice to maybe have like an option of like, “Oh, she’d rather be with a Girl/Woman”. (Participant 26, Intervention, Girl/Woman, 16, table 3 for expanded quote).

At the same time, participants in both study conditions detailed experiencing a tailored, individualized approach to care to meet their needs. Components of these approaches, as described by participants, included goal setting; identifying solutions to challenges; and self-care strategies and techniques to practice at home.

She encouraged a healthy lifestyle …. She gave me a lot of options … That’s what I wanted … I am definitely seeing improvements in myself and my family sees it. (Participant 42, Control, Girl/Woman, 16, table 3 for expanded quote).

Participants in both study arms detailed how health care providers were flexible with the care plan based on their evolving needs. Youth described how they felt seen, heard, and not rushed during the sessions. Participants noted that this approached made them more willing to open up and speak about their issues.

Like, I definitely wanted somebody who would, like, understand me and everything, and when I went in for my, like, first appointment, like, they were super friendly, and they were very understanding, and supportive of, like, my opinions, and they supported me no matter what happened. Which, I – That’s what I really wanted, which I felt was definitely helped me with my confidence, like, gave me a bit of a boost in confidence, which was awesome. (Participant 19, Intervention, Girl/Woman, 17 years).

There were some youth within the Control group who did not experience a tailored approach to care, despite an expressed preference for this approach, indicating its importance in youth services. According to the chart review, these participants had one or two visits with the provider. These youth described perceiving that the provider was not interested in their wellbeing or ensuring they were comfortable during the session. Participants described feeling disconnected from the service provider, which contributed to feeling uncomfortable, not listened to, and judged. For some, these feelings contributed to the discontinuation of care.

Like, he just kind of got a background … but, nothing really ever came from it. There wasn’t any like coping method … I’m glad, like, that I didn’t have to go back and see him. (Participant 17, Control, Girl/Woman, 15, table 3 for expanded quote).

#### Supportive setting

A supportive setting articulated factors related to comfort, as they affected the youth’s ability to feel valued and engaged in the services. These factors included friendliness of health care providers and reception staff, wait times, the consequences of missing appointments, service hours and location, comfortability of the service environment, and self-stigmatizing attitudes. Participants reported that experiencing a supportive setting contributed to their satisfaction and improved mental health outcomes. In both study groups, participants described reception staff and service provider friendliness as experiencing positive attitudes, understanding, patience, and kindness. This approach made youth feel more comfortable, confident, safe, open, and helped establish trust with the services. Within service settings, participants described how they felt welcomed, calm or at ease, as well as a sense of belonging. Participants attributed these feelings to a quiet and calm environment, not feeling rushed, and being able to experience care at their own pace.

like when I did meet my counsellor it was a really like, it was a really friendly environment. It wasn’t – It is formal, but it feels like a very safe space for me to talk about, like, what like I’m feeling and like how I can like make my life a little bit easier, and kind of certain obstacles for myself. It was just very like, better than I expected. (Participant 6, Intervention, Girl/Woman, 16 years).

Participants in both study arms acknowledged several challenges to accessing services, including service hours and location. Youth reported that they were restricted in scheduling appointment times due to busy provider schedules. They expressed frustration as it meant that they had to miss school or extracurricular activities.

It’s kind of contradictory, because I had stress levels about missing school and I had to miss school to go talk to about my stress levels about missing school. (Participant 25, Control, Girl/Woman, 14 years, table 3 for expanded quote).

Similarly, participants described the location of the services as inconvenient and hard to reach, with slightly more youth at the Control sites reporting these barriers to care. Participants in the Intervention group shared how location adversely affected their access and continuity of care.

Well I mean, with depression sometimes you don’t feel like getting up and going places, … it was just too far and in my head I was thinking “well, it’s not worth it”. (Participant 1, Intervention, Girl/Woman, 17 years, table 3 for expanded quote).

Control group participants experienced lengthy wait-times and the lack of availability of services. They described waiting months to make initial and subsequent Control group appointments. Participants described uncertainty and frustration about their appointment, detailing how they were annoyed at the lack of communication and follow-up.

It is pretty annoying that I can’t schedule anything if I need it right now. Like I need to plan ahead my mental breakdowns. (Participant 18, Control, Girl/Woman, 16, table 3 for expanded quote).

Other participants shared that they called the service but did not receive a callback. Several participants in the Control group described having a hard time remembering appointment dates, expressing a preference to receive service reminders (either by text, email, or regular mail) a few days in advance.

Well, I was pretty pissed because they didn’t even call me to remind me about the appointment … Like I had completely forgot. (Participant 18, Control, Girl/Woman, 16 years old, table 3 for expanded quote).

These factors contributed to treatment delay. Participants in the Control group were frustrated at having to wait months to see the service provider, while dealing with their mental health concerns. These participants described an unmet need for immediate care. Within the Control group, participants were financially charged for missed appointments, which prevented them from continuing care due to financial constraints.

Due to these delays in services and the need for care, youth sought services elsewhere, outside of Control group referrals. Youth at the Intervention sites described experiencing shorter wait times than expected and feeling satisfied with service wait times.

In both study arms, participants liked how the facilities were furnished and decorated. These details included paintings and posters on the wall, comfortable seating, natural light, and an open reception area. Participants in the Intervention group liked the board games, trendy furniture, brightly painted walls, and fidget toys. Meanwhile participants in the Control group liked the family-friendly, clean and professional look of the environment.

Participants in the Control group experienced self-stigma towards seeking and using MHSU services within the hospital setting. They were of the opinion that they would not receive the help they needed and that the hospital was for individuals with more severe mental health concerns. Some participants described feeling uncomfortable and not welcome because of the hospital setting. It also contributed to some participants leaving the service.

Like I was pretty sure that like I wouldn’t really receive like the help that I really wanted at a … hospital. So I was kind of expecting it. (Participant 3, Control, Girl/Woman, 16).

Other youth acknowledged how they initially were wary of seeking MHSU services at the hospital, however, their attitudes changed once they experienced services that provided comprehensive information and a tailored approach to care.

Well, it made me feel more welcome … Like, before I started going there, I thought it was weird. (Participant 8, Boy/Man, Control, 15, table 3 for expanded quote).

A few participants in the Intervention group expressed stigmatising attitudes at not wanting to be seen accessing the services. As such, these attitudes decreased their interest to engage in MHSU care.

## Theme 2. Autonomy

Participants in both the study arms described experiencing autonomy in care, or having the knowledge, space and feeling empowered to make their own health care decisions. This theme was described as participants’ level of involvement in decisions related to their own care; and the degree to which they were able to choose their caregiver’s involvement.

### Subtheme 1. Youth voice and choice

Participants described how they were involved (or not) in decisions related to their care within the services. In both study arms, participants’ level of involvement were described as how participants made all of the care decisions by themselves; shared the decision-making with caregivers and/or providers; or were not involved in decisions about their care at all. A greater level of involvement in decision-making facilitated satisfaction among participants and the ability to express their own needs.

I think that for the first time I felt that I was making decisions that would benefit me … It was like, “What do I want out of this? What can I do myself to help me feel better?”. (Participant 16, Intervention, Girl/Woman, 16, table 3 for expanded quote).

Participants who made decisions by themselves were encouraged by the service provider to exercise their autonomy. These decisions included the services they wanted to access (e.g., psychiatrist) and the delivery of services (e.g., group versus individualized therapy). It also included defining how they wanted the sessions to be organized, what they wanted to talk about, and their thoughts on medication.

Some participants in the Control group expressed a lack of involvement in decisions related to their care plan. These youth described how service providers did not involve them in deciding on options for care or when to end sessions. Their lack of involvement led to frustration and distress among participants at being left out of their own care.

… It was a one appointment thing. I was told I have a good head on my shoulders and that I’ll be just fine. I wasn’t really referred … and I wasn’t really given, like, ways to help control it. (Participant 25, Control, Girl/Woman, 14 years of age, table 3 for expanded quote).

For participants who described shared decision-making with service providers, these decisions included discussing different service options, treatment plans, and compromising on their care plan. When youth described shared decision-making with caregivers, descriptions focused on not having enough experience with services and preferring caregiver support to assist them in the decision-making process.

Like I don’t have much experience with dealing with like doctors … so I usually ran it through my mom first. (Participant 31, Intervention, Girl/Woman, 16, table 3 for expanded quote).

Participants in both study arms described how making decisions contributed to feelings of empowerment, independence, and control. Participants shared how making decisions empowered them to communicate when the plan was not working. Some participants shared that this experience was their first time making their own decisions. Being able to make these decisions contributed to feeling more comfortable with themselves, speaking their mind, and not being afraid to ask questions when confused.

It made me feel like I wasn’t on a downward spiral. Like I actually had a chance to make myself feel better. (Participant 36, Intervention, Boy/Man, 17, table 3 for expanded quote).

It also contributed to continuity of care, in that some youth described how they would not have engaged in a program if their input had not been considered.

#### Choice in caregiver involvement

Participants detailed the degree to which they were able to choose (or not) the level of involvement of their caregiver in their service experience. In both study arms, some youth described caregivers as acting as navigators to their care, including scheduling appointments, guiding the youth during the initial appointment, filling out paperwork, and facilitating drop off and pick up at sessions. Some participants within both groups described how it was their decision and preference to involve their caregivers. For those who preferred to have their caregivers involved, this involvement centered on needing support and feeling more confident during the session with the caregiver present. Some youth in both study arms described it as healing the relationship with the caregiver, while others wanted the caregiver involved so that they could receive support at home.

I think it would be more helpful to have my parents, like, involved …. (Participant 19, Intervention, Girl/Woman, 17, table 3 for expanded quote).

For participants who did not want their caregivers involved, descriptions centered on privacy, feeling uncomfortable, and a need for independence. Some participants in both conditions detailed caregiver dominance in their service experience, in that the caregiver would attend all of the appointments, control the discussion with the provider, and act as the point of contact outside of services. They also shared that their caregivers opposed what they would say during the session, adversely influencing their treatment plan.

And then when I went to [the Intervention site] with her … like I said I was seriously struggling … and then my mom, like, said that like ‘cause I don’t do any work … So it was a bit hard. It wasn’t the nicest because … It–it’s not – there’s not really a lot of excuses for the way I feel in a way? It’s not normal, so, yeah. It just wasn’t really nice. (Participant 26, Intervention, Girl/Woman, 16 years of age, table 3 for expanded quote).

Yeah, of course. Like by my psychiatrist, yeah, but like not really with my mom though. But then like sometimes like my mom wouldn’t want me to take medications, right, so then it would like affect me … well it just affected me because like I would stop taking them like eventually right, so … yeah. Like it made me like feel like anxious because like I knew that like my Mom would kind of have like control over, like, if I went or not. Cuz then she would be the first to know about it. (Participant 29, Control, Girl/Woman, 15 years).

Participants who experienced caregiver dominance felt distressed and expressed that their decisions and privacy were not respected. These youth were confused as to who made the decision to involve the caregiver: the caregiver or the service provider.

## Discussion

This study is the first qualitative study embedded within a pRCT to explore experiences of an ICCT service model and standard hospital-based (or delivered) care globally. Overall, findings indicate that participants in both the intervention and control arms reported feeling valued and respected when they received tailored, consistent services; comprehensive information; developed a supportive, collaborative relationship with the service provider; and had opportunities to use their voice in their care plan. When participants perceived that their basic needs were met, they reported greater satisfaction, improvement in mental health status, and usefulness of services compared to when their needs were not met. These findings indicate the importance of providing services that maintain youth’s dignity, respect, and autonomy, within the framework of youth-centered or youth-friendly care. Our findings also add to the limited global research on integrated service models for youth within real-world settings.

**Fig. 1:**
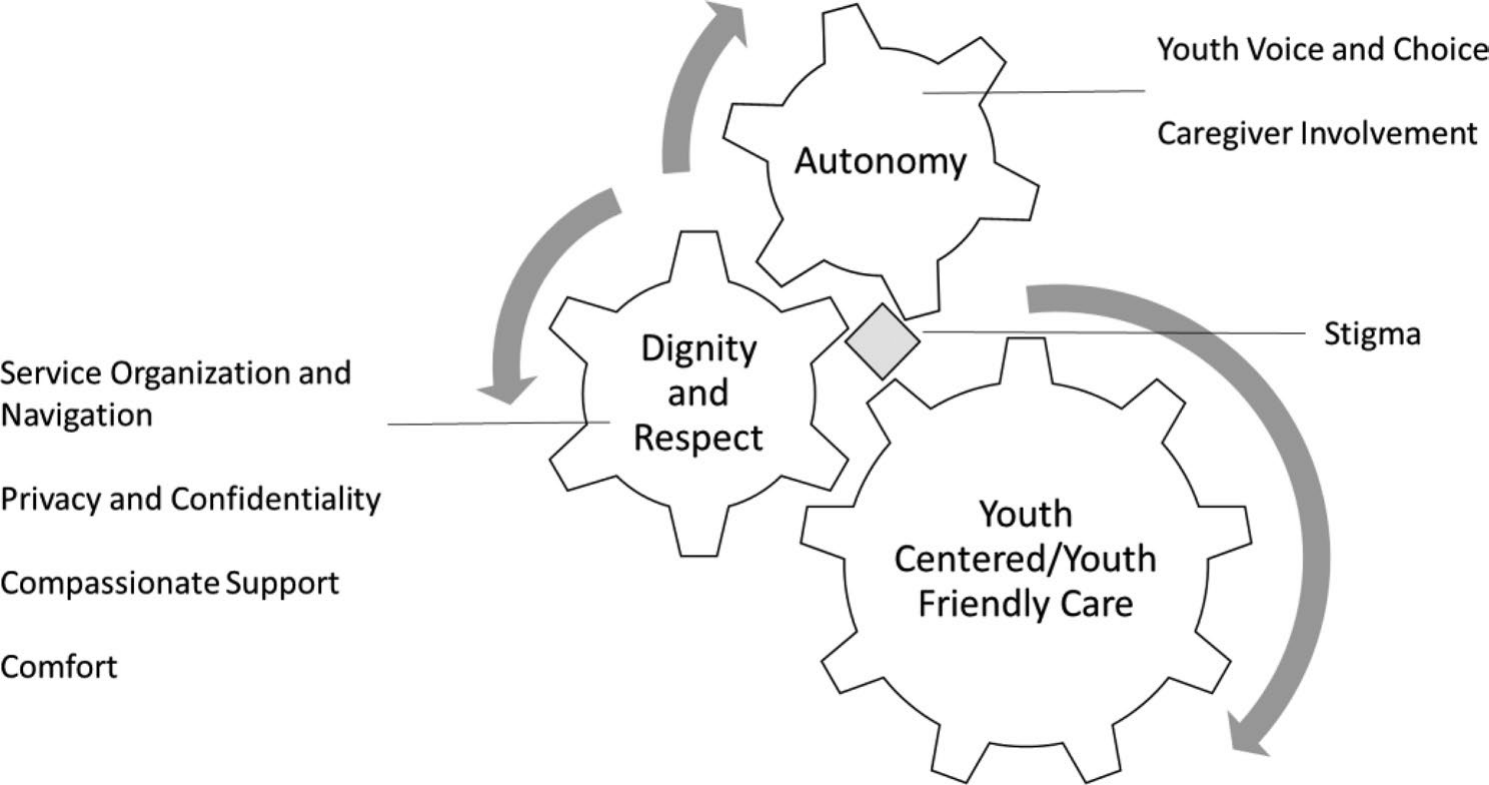
Themes and Subthemes on Youth’s experience with integrated collaborative Care teams vs treatment as usual

We developed a framework of the aspects of youth-centered care, based on the themes and subthemes identified within the study (Fig. 1). The model considers the interaction of characteristics of dignity, respect, and autonomy as influencing youth-centered care.

Dignity and respect are complex, multi-faceted concepts not well defined or explored in youth MHSU services [47, 48]. Within the World Health Organization’s Mental Health Action Plan, dignity and respect for human rights are essential for accessible, culturally appropriate, person-centered, and high-quality health care [49, 50]. Dignity includes dimensions of value and worth that are felt both internally and socially [51], while respect represents the social and behavioural actions that contribute to dignity [52–54]. Dignity and respect are preserved in mental health services when individuals experience privacy, confidentiality, communication and information, active participation, and decision-making in their own care [50]. Our findings align with prior limited evidence on youth’s experience of dignity in inpatient hospital settings, wherein youth defined dignity in terms of privacy; respect; autonomy; and friendly and effective communication with the provider [47, 55, 56]. Similar findings were shown in a recent systematic review of dignity among pediatric populations [57]. Meanwhile, autonomy is a dynamic process in which youth have the knowledge, space, and are empowered to express their opinions and views freely [58]. Aligned with previous research [59], our findings illustrate the importance and need for autonomy in services and independence during this phase of life. Dignity, respect, and autonomy enhance recovery and satisfaction, empower, and provide youth a sense of confidence [51]. Silverstein and colleagues (2024) [57] developed the ReCAP (Respect, Communication, Agency and Autonomy, and Privacy) conceptual model for pediatric patients. The model provides a flexible definition of dignity, in which it is co-created by patients, clinicians, and caregivers. It focuses on dignity as a human right, person-centered, and an intrinsic quality. Discussions on dignity should occur within research, medical education, and at the bedside.

Dignity, respect, and autonomy are basic needs and tenets of youth-centered, or youth-friendly, care [51]. Youth-friendly MHSU care is defined as the provision of holistic, integrated services that respond to youth needs; ensures that youth feel valued and respected; and offers choice and a partnership approach [60]. These concepts are also fundamental to healthcare ethics [61]. Given the limited global evidence on dignity and respect in youth MHSU services, our findings suggest a need to not only develop and standardize a definition of dignity and respect in these services, but also methods that measure dignity and respect in youth MHSU care. Based on our findings, this definition could include aspects related to privacy and confidentiality; comprehensive information; coordinated care; and provider commitment and engagement. These concepts are influenced by youth’s social, cultural, and economic contexts [62]. As such, the definitions and measures should be adaptable to contexts and intersectional identities. Further research is needed on youth’s perceptions and concerns about dignity and respect; if these perceptions change as they age; how they are influenced by their contexts; and how these concepts are associated with youth MHSU outcomes [63]. Research on these concepts in other global, integrated service models should be conducted.

Youth are vulnerable to experiencing a greater loss of dignity and respect in health services compared to early-to-mid adulthood [63, 64]. They also experience a loss of autonomy when they are prevented from accessing the care they want and need, which is associated with feeling a loss of worth [65]. Despite the Comprehensive Mental Health and Addictions Strategy in the province of Ontario, Canada, where the study took place, *Open Minds, Healthy Minds,* which promotes the principles of dignity, respect, and autonomy [66], as well as *Moving on Mental Health: A System that Makes Sense for Children and Youth* [67], the findings illustrate the importance of providing information and resources to youth about services, as well as coordination of care to contribute to youth’s experiences of dignity, respect, and autonomy.

Based on the findings of the study, ICCT services may be complementary to hospital-based services. It will be important to ensure that integrated models are adequately funded to fully embed specialized MHSU services, as also suggested by findings of this study. There is also a need for greater investment of financial resources earmarked for community MHSU services to ensure sustained, expert, and integrated care for youth with more substantial and evolving needs.

Potential pathways to improve dignity, respect, and autonomy should involve developing an integrated or “systems” approach to organizing community-based and hospital-based youth MHSU services. This would help to ensure seamless transitions across services of varying levels of intensity, so youth do not fall through the cracks [27]. There are emerging examples of this with Youth Wellness Hubs Ontario, an integrated youth service model partially inspired by YouthCan IMPACT [26]. Youth and caregivers should be engaged and involved in service design and delivery. This study demonstrates the feasibility and value of youth and caregiver engagement in service design and delivery. For example, youth emphasized the importance of shared decision-making and youth who were involved in shared decision-making highlighted the value of this approach. Recently, there has been substantial expansion of youth and caregiver engagement in research. For example, the International Network for Research Outcomes in Adolescent Depression Studies (IN-ROADS) engages youth and caregivers in the co-development of a core outcome set for adolescent major depressive disorder clinical trials [68]. Further, identifying measurable performance targets to ensure timely, equitable access and culturally appropriate care for youth is required across both hospital and community-based MHSU service settings [69–72]. There is also a need to develop evidence-informed guidelines for youth-friendly practices and educational materials that promote dignity, respect, and autonomy in MHSU services [73]. These resources should be embedded within psychiatric training and professional practice [69, 74].

Stigma continues to be a barrier for MHSU services. Defined as a dynamic social process, stigma is reinforced, maintained, and promoted by those with more political, economic, and social power in society. Stigma maintains this power structure, underscoring differences between groups [75]. To address stigma, services could promote socialization opportunities within the community between those who do and do not have lived experience (e.g., peer support and co-design of anti-stigma programs), as recent evidence showed that increasing this contact reduced stigma [76].

This study has a number of limitations. The study was conducted in an urban setting in a high-income country, and therefore the findings may not be applicable to other settings (e.g. rural areas). The urban setting includes a high density of specialized mental health providers, limiting the relevance for rural or remote areas, where resources are typically less available. This study did not test causal mechanisms of change between intervention and control. Further research needs to be conducted on the causal mechanisms of change between community-based and hospital-based services. In addition, this study only reflects the views of adolescents; the issues and experiences of young adults receiving services in community and hospital-based services may vary considerably. Youth in this study identified varied ancestry/ethnic group or cultural backgrounds, though the majority identified as White. Moreover, youth were asked about their experiences of culturally-appropriate or -specific services. It will be important for future research to expand the emerging evidence-base on the experiences of Indigenous, Black, and racialized youth and caregivers [77–84]. Our sample included a majority of youth identifying as girls/women. Future research should intentionally investigate the experiences of boys/young men and gender diverse youth. The interviews took place over the phone. Although the research team tried to make the semi-structured interviews as natural as possible, this technique could have influenced the results. For example, there could have been connectivity issues and nonverbal cues not observed. By design, this study examines youth perspectives – other individuals in these interactions (e.g., caregivers, clinicians) may have different perspectives. Future research should examine these perspectives.

## Conclusions

To our knowledge, this study is the first qualitative study embedded within a pRCT investigating youth’s perceptions and experience of receiving an integrated collaborative care team model of service delivery or treatment as usual in hospital settings. It adds to the limited evidence base on dignity, respect, and autonomy within MHSU services. Determining how youth experience services in community-based and hospital settings is critical as it can help guide service change and transformation, and reduce treatment gaps. Based on youth’s experiences, factors that promote dignity, respect, and autonomy are critical to promoting positive service experiences and improved MHSU outcomes.

## Electronic supplementary material

Below is the link to the electronic supplementary material.

Supplementary Material 1

Supplementary Material 2

## Data Availability

The datasets used and/or analysed during the current study are available with permission from each participating study sites.

## Abbreviations

CAMH: Centre for Addiction and Mental Health
ICCT: Integrated Collaborative Care Teams
IN-ROADS: International Network for Research Outcomes in Adolescent Depression Studies
IYS: Integrated Youth Services
MHSU: Mental Health and Substance Use
MGH: Michael Garron Hospital
NYGH: North York General Hospital
pRCT: Pragmatic Randomized Controlled Trial
SFBT: Solution-focused brief therapy
SHSC: Sunnybrook Health Sciences Centre
SickKid: The Hospital for Sick Children
