# Supplementary material for: A qualitative study exploring youth’s experiences of hospital- and integrated community-based mental health services: the YouthCan IMPACT initiative
Source: BMC Psychiatry. 2025 Nov 12;25:1084. doi: 10.1186/s12888-025-07523-7 (PMC12613439; doi:10.1186/s12888-025-07523-7)
Supplement: Supplementary file 1 — Supplementary Material 1 [file 12888_2025_7523_MOESM1_ESM.pdf]

YouthCan IMPACT
Interview Questions for Youth – Semi Structured Interview Guide

**Please note that since this is a semi-structured interview, not all questions will be asked in the order outlined below. Prior to beginning the interview, the interview checklist must be completed, consent obtained and an introduction to the study provided.*

General Introduction

Warm up and introductions.
Do you have any questions?

YouthCan Timeline and Overall Experience

Let's start at the beginning.

1) Tell me about the first time you came to _______ (ICCT name or hospital)?
   Probes: When *did you come?*
   Do you recall ***who came with you****?*
   What were your ***first impressions****?*

2) Tell me about your experiences using services at_______ (ICCT name or hospital)?
   Probes: *What did you* ***like****?*
   What ***didn't you like****?*
   If you could go back and ***do it all over again****, what would you like to be different?*

3) Was there anything you found **most helpful** about the services? **Least helpful**?

4) When was the **last time you used** services at (ICCT name or hospital)?

5) Are you **planning to go back** for more services? Why or why not?

Access to Services

Let's talk about your thoughts on getting started with services and accessing them when you needed to.

1) Can you tell me how it went for you **getting started** with services at (name of ICCT or hospital)?
   Probes: *Were you seen* ***more quickly*** *than you expected?*
   Did it take ***longer*** *than you expected to get started?*
   Was the process ***straightforward****?*
   Was it easier or more difficult than you expected?
   Not including the initial research assessment, did you have to ***repeat your story*** *to multiple people?*

2) Can you tell me about **getting services when you needed them**?
   Probes**:** *Did you have to* ***wait long*** *to be seen when you came here?*
   Did you feel ***well-informed*** *about how to access services?*

*Were you able to **access more than one kind** of service in one place?*
*Was it easy or difficult to **access services when you needed to**?*
Did you receive the types of services you needed when you needed them?
*What happened when you **missed an appointment**?*
*Did services **conflict with school** or other activities?*
*Were services **flexible**?*

3) What were your expectations going in for what types of **service providers** you would see, and how did that compare to who you actually ended up seeing?
Probes: *What types of service providers did you work with? **Psychiatrist? Counselor? Nurse**?*
*Were you **happy with the types** of service providers you saw?*
*Was it **easy to get linked** up with certain types of providers?*
*Is there any other type of service provider you would have **liked to have seen but didn't**?*

<u>Service Setting and Connection</u>

I'd like to hear about the actual environment where you received services.

1) What did you notice about the **space**?
Probes: *Did you find the space **welcoming** for you and other people?*
What made it welcoming or unwelcoming?
*Was the **vibe friendly** for youth?*
*What did you **like** about the space?*
*What did you **not like** about it?*
*Did you feel **comfortable** in the space, like it was a **safe** space?*
What would have made it better?
*How does it **compare to other places** you've received services or health care?*

2) What did you think about **the location** of (name of ICCT or hospital)?
Probes: *Were you **satisfied** with the location?*
*What was **convenient or inconvenient** about the location?*
What would have made the location better for you?
*Were all the services you received located in **one place**?*
Did you have to seek services elsewhere? If so, what was that like for you?

3) How did you feel coming into this the environment?
Probes: *Did you know **where to go** and what the **process was when you arrived**?*
*Did you find the **staff welcoming**?*
*Did you have a sense of **belonging** in (name of ICCT or hospital)?*
What gave you this sense of belonging, or what prevented a sense of belonging?

<u>Fit of Services, Integration and Holistic Needs</u>

1) I'd like to hear your thoughts about the services you received. Tell me what you thought about **the fit between the services you received and what you were looking for**.
Probes: *Did the services address what you were looking for help with?*
*Were they a **good fit** for your needs?*

Do you wish you had received different services?
What services would you like to have received but didn't?

2) Can you tell me how **satisfied you were with all the services you received as a whole**?
Probes: *What types of services did you receive?*
Did you receive a set of services from different providers or just one provider?
Did you wish you received more types of services?
If you received services from multiple providers, were these services coordinated?
Did these providers ***work together like a team****?*
Did you feel that these services ***addressed all of your needs rather than just one specific*** *piece?*
Were there any services ***you didn't expect to receive that you found helpful****?*

3) What was your experience like working with the staff at (name of ICCT or hospital)?
Probes: *Were they* ***friendly and welcoming****?*
Were they ***responsive*** *to your needs?*
Were they ***flexible****?*
Did they seem to ***adapt their approach to what you needed****?*

Empowerment, Engagement and Parental Involvement

1) Can you describe for me what your experience was like with **making decisions** about your treatment at (name of ICCT or hospital)?
Probes: *How* ***much of a say*** *did you have in making decisions about your treatment here?*
What ***kinds of decisions*** *did you make?*
When you think about your health (mental and physical), ***who makes most of the decisions****?*
Does your service provider or parent make most of the decisions?
What kind of decisions do you make about your health in general? Was that the same or different here?

2) Describe for me **how your input was part of making decisions about your treatment**.
Probes: *Did you feel like your input was valued in making decisions about your treatment or the types of services you received?*
Did it seem like you had a ***choice about your treatment plan*** *or the types of services you received? Did you feel like* ***your wishes were heard****?*
Did you feel like you played an ***active role*** *in your treatment?*
How did you know whether your input was taken into consideration?
Did service providers hear and understand your concerns*, and if so, how did you know?*
Did you feel like you had a ***variety of options*** *to choose from and that these were well-explained to you?*

3) Tell me about the involvement of **your parent or caregiver** in your services at (name of ICCT or hospital).
   Probes: *Was a parent or **caregiver involved** in your experience?*
   How *were they involved?*
   *Did your **service provider have direct contact** them?*
   Why were they involved or not involved?
   What was the process of getting them involved?
   *How were **you involved in the decision to involve them**?*
   Did you think that it was helpful or would have been helpful for them to be involved or not involved?
   *Was your parent or **caregiver well-informed about the services available to them**?*
   *Were there any **additional supports** that would have improved your parents' or caregiver's involvement?*

4) To what extent did you feel prepared for **next steps** at the end of your appointments?
   Probes: *Did you understand what the next steps would be?*
   *Did you know **who to contact if something came up** before your next appointment?*
   Did you know your service provider's name?

<u>Conclusion and Thank You</u>

1) In an **ideal world**, what would services look like for you?
2) If a friend of yours needed support, what would you say about (name of ICCT or hospital)? What services would you **recommend** to them?
3) Is there anything else we haven't covered that you would like to add or think we should know?
4) Do you have any questions?
