# Supplementary material for: A qualitative study exploring youth’s experiences of hospital- and integrated community-based mental health services: the YouthCan IMPACT initiative
Source: BMC Psychiatry. 2025 Nov 12;25:1084. doi: 10.1186/s12888-025-07523-7 (PMC12613439; doi:10.1186/s12888-025-07523-7)
Supplement: Supplementary file 2 — Supplementary Material 2 [file 12888_2025_7523_MOESM2_ESM.pdf]

Additional File 2. List of Exemplar Quotes

Themes	Subthemes	Exemplar Quotes
Dignity and Respect	1. *Service organization and navigation*	It's never really been hidden from me at all how things work around here, which I really appreciated. That helps a lot with establishing that trust. But I just feel super comfortable being here and talking to people. (Participant 14, Control, Female, 15).<br>Well I had meetings with the social worker, and we outlined all the services and programs they have there. And the different methods, everything that I really needed to know. And then I could pick from there which services I wanted to go forward with (Participant 21, Intervention Group, male Boy/Man, 16 years of age).<br><br>When we were going over what services I would be put into, they gave me a whole list, like a whole list of programs and what they cover. So my provider felt that it would be better if I was put into a group therapy that goes over, like basically depression and anxiety. And so far, my experience in going to that service has been just every– Like it's just gone over everything that I've been trying to work on over the past couple years (Participant 5, Control group, female, 14 years of age).<br><br>I always knew what my next steps were `cause they were obviously– it felt like they were told very clearly, everything was clear on what I needed to do. And it– it made me feel– it definitely made me feel like that I– that I was being informed and given the right information of what I needed to do. (Participant 22, Intervention, Boy/Man, 16).<br><br>When we were going over what services I would be put into, they gave me a whole list, like a whole list of programs and what they cover… And so far, my experience in going to that service has been just every– Like it's just gone over everything that I've been trying to work on over the past couple years. It made me very happy considering that it's been a long time that I've been trying to find, like- trying to find a program that would fit these needs… and [this hospital] just had that program … (Participant 5, Control, Girl/Woman, 14).
	2. *Privacy and Confidentiality*	Well, they've been very understanding about my parental situation at large. So it's kinda been a thing where they were happy to get me on my medication and everything and not contact anyone about it. Obviously, it's no one else's business really. (Participant 14, Control, Female, 15).

	3. *Compassionate Support*	Just because like I've never been anywhere else and that was the place where I went to for a couple of months, weekly. Well, like, it really helped with my depression. It helped a lot. Because I was probably depressed for like a year, and now I feel like more like myself. (Participant 4, Intervention, Girl/Woman, 17).<br><br>I think, maybe, just… In my experience, I think they could've listened more and not tried to, just, put their opinions over mine. (Participant 9, Intervention, Boy/Man, 14).
	Comfort	
	Provider Commitment	What I was looking for, like I said, was ways to deal with my stress. `Cause it was like really bad… And honestly, it's improved a lot now. It's like rarely it, like, affects my everyday life now ... I definitely got what I expected. (Participant 38, Intervention, Girl/Woman, 16)<br><br>It was what I needed, because I had been on medication but it wasn't strong enough and it wasn't helping me at all. And I couldn't really function at all at the time. But after getting the help I needed, it really like, it made me so much better. (Participant 18, Control, Girl/Woman, 16).<br><br>When I was talking about my problems he was like, not really like– Like it wasn't helpful, like, like his solutions. It wasn't really for me, like… I don't really think he– he really understood what I was trying to tell him about or really what I was talking about… that was kind of, like, something I didn't' really like. Like it wasn't really helpful the advice, that he was giving me or it wasn't practical. (Participant 3, Control, Girl/Woman, 16).
	Supportive Setting	Oh yeah. I'd say that all the workers there were just friendly and welcoming as a whole. Like everyone kind of made an effort to make you feel like you belong ... It made me feel more inclined to go to my, to go to my appointments. (Participant 37, Intervention, Girl/Woman, 17)
Autonomy	Youth Voice and Choice	I got to choose what to talk about with them and I would– I could ask for tips. I felt comfortable asking for things from them, like, ways to cope with certain things and stuff like that. (Participant 32, Intervention, Girl/Woman, 15)

		It made me– `cause like I was, I guess in control. Like It was up to me. My choice… I can choose to take whatever steps I wanted to, to improve myself. (Participant 38, Intervention, Girl/Woman, 16)<br><br>All of it actually. I think they actually told me that too, that I had all the decisions in what actually I'm getting and everything. (Participant 41, Control, Girl/Woman, 16)<br><br>Yeah, I had a say to the extent that, like, my psychiatrist recommended this and then told me– I'm– I'm thinking about section 23 schools here. So, my psychiatrist was like, "Hey, there's a school here, this is what it's all about. Do you think you'd want to look into that?" I said yes, and so she really tried to make my decision count, I guess. (Participant 13, Control, Boy/Man, 16)
	Choice of Caregiver Involvement	They [caregivers] definitely put added feedback– Like, they would say their side and, like, I would hear their side and everything, and we would try to come up with ways to, like, you know, find the middle ground. (Participant 19, Intervention, Girl/Woman, 17)<br><br>So yeah. I guess my decisions were– Well my– I don't even think my parents- My parents didn't really help me make any like, medical decisions. (Participant 22, Intervention, Boy/Man, 16)<br><br>I don't want her to be involved and she's fine with it. She's fine with it, yeah. (Participant 27, Control, Girl/Woman, 16)
Stigma		… I guess for me the scariest thing was gonna run into, I guess, someone that I knew. So I was a little anxious about doing– like going there `cause I didn't want people being like, "Oh, what's…", you know, "what's wrong with this person?" or whatever. (Participant 22, Intervention, Boy/Man, 16)<br><br>I didn't really like being in a hospital, like that was the only part that I didn't really like. Like, I feel like if it were somewhere else, it would have been, like, a bit better but, like, just being in a hospital just, like, made me really uncomfortable... (Participant 34, Control, Girl/Woman, 16).

		Because at the end of the day like I don't think like the majority of people that get referred to … hospitals are people who really need to get this kind of service… for me at least, it was kind of really unnecessary (Participant 3, Control, Girl/Woman, 16).
